# Does titanium in ionic form display a tissue-specific distribution?

**DOI:** 10.1007/s10534-016-9930-8

**Published:** 2016-04-04

**Authors:** Magdalena Golasik, Pawel Wrobel, Magdalena Olbert, Barbara Nowak, Mateusz Czyzycki, Tadeusz Librowski, Marek Lankosz, Wojciech Piekoszewski

**Affiliations:** Department of Analytical Chemistry, Faculty of Chemistry, Jagiellonian University in Krakow, Ingardena 3, Krakow, 30-060 Poland; Faculty of Physics and Applied Computer Science, AGH University of Science and Technology, al. Mickiewicza 30, Krakow, 30-059 Poland; Department of Radioligands, Faculty of Pharmacy, Medical College, Jagiellonian University in Krakow, Medyczna 9, Krakow, 30-688 Poland; Department of Pharmacobiology, Faculty of Pharmacy, Medical College, Jagiellonian University in Krakow, Medyczna 9, Kraków, 30-688 Poland; DESY Photon Science, Notkestraße 85, 22607 Hamburg, Germany; School of Biomedicine, Far East Federal University, M 715 Office, Bldg. 25, Ajax, Vladivostok, Russky Island Russia 690922

**Keywords:** Titanium, Organ distribution, Rat tissues, Micro synchrotron radiation-induced X-ray fluorescence

## Abstract

Most studies have focused on the biodistribution of titanium(IV) oxide as nanoparticles or crystals in organism. But several reports suggested that titanium is released from implant in ionic form. Therefore, gaining insight into toxicokinetics of Ti ions will give valuable information, which may be useful when assessing the health risks of long-term exposure to titanium alloy implants in patients. A micro synchrotron radiation-induced X-ray fluorescence (µ-SRXRF) was utilized to investigate the titanium distribution in the liver, spleen and kidneys of rats following single intravenous or 30-days oral administration of metal (6 mg Ti/b.w.) in ionic form. Titanium was mainly retained in kidneys after both intravenous and oral dosing, and also its compartmentalization in this organ was observed. Titanium in the liver was non-uniformly distributed—metal accumulated in single aggregates, and some of them were also enriched in calcium. Correlation analysis showed that metal did not displace essential elements, and in liver titanium strongly correlated with calcium. Two-dimensional maps of Ti distribution show that the location of the element is characteristic for the route of administration and time of exposure. We demonstrated that µ-SRXRF can provide information on the distribution of titanium in internal structures of whole organs, which helps in enhancing our understanding of the mechanism of ionic titanium accumulation in the body. This is significant due to the popularity of titanium implants and the potential release of metal ions from them to the organism.

## Introduction

The ongoing ageing process of the population in many countries will lead to the growing demand for restoring the function of damaged organs and tissues (Holzapfel et al. 2013). Medical devices designed for such purposes can be made from a variety of biomaterials, but one of the most popular are Ti-based alloys (Geetha et al. 2009). Many studies reported that titanium implants undergo biodegradation in the body (Matusiewicz 2014; Cundy et al. 2015; Martín-Cameán et al. 2015). Metal ions and biomaterial debris are released into peri-implant tissues as a result of corrosion and after this they can be transported by blood [Ti(IV) ions bound to transferrin] to other biocompartments (tissues) (Soto-Alvaredo et al. 2014; Zierden and Valentine 2016). But some studies reported that metallic debris did not migrate deep into the tissue around implant (Guibert et al. 2006) or the leakage of Ti to adjacent bone and later to organism was limited (Passi et al. 2002).

Up till now, most studies have been focused on the biodistribution of titanium(IV) oxide as particles in organism (Geraets et al. 2014; Elgrabli et al. 2015). But, as Sarmiento-González et al. (2009) suggested, soluble Ti ions are the main degradation products of these metal implants. Moreover, metal in such form has shown higher toxicity to cell lines (human enterocytes and murine osteoblasts) than the nanoparticles at similar concentration (Soto-Alvaredo et al. 2014). The health effects of long-term exposure to ionic titanium are still not well-described.

One aspect of potential risk assessment attributed to this form of metal is to evaluate places of its accumulation on the level of organs and their internal structures. There are many imagining techniques that can be used for that purpose including scanning electron microscopy with X-ray microanalysis (SEM/EDS) (Patri et al. 2009; Zhang et al. 2015), particle-induced X-ray emission spectroscopy (PIXIE) (Sugiyama et al. 2015) or laser ablation-inductively coupled plasma mass spectrometry (LA-ICPMS) (Davies et al. 2015). Another popular method that is used for the visualization of metals distribution in whole organs is X-ray fluorescence (XRF). It possesses many advantages such as nondestructiveness and simple preparation process (Ralle and Lutsenko 2009). An exciting X-ray beam can be produced either with an X-ray tube or with a synchrotron storage ring. Synchrotrons are a common choice for the investigation of biological samples where the elemental concentrations are very low. They offer very high intensities, which increases the sensitivity and decreases the detection limits, and good resolution (down to 1 µm) (Lobinski et al. 2006). In previous studies micro synchrotron radiation-induced X-ray fluorescence (µ-SRXRF) was used for in vivo investigation of distribution and accumulation of nano-TiO_2_ in lungs (Zhang et al. 2013, 2016), the olfactory bulb (Wang et al. 2007) and brain (Wang et al. 2008) of mice after intratracheal instillation. As far as we know, no study of Ti imaging in whole organs for the analysis of biodistribution of titanium in ionic form has yet been published.

In this study, we presented the first quantitative assessment of the spatial distribution of titanium in kidney, liver, and spleen collected from rats administered intravenously or orally with soluble salt of this metal. We utilized a µ-SRXRF technique for bioimaging of titanium in whole organs with quantitative data. Additionally we evaluated whether Ti influences the distribution of other metals (Ca, Cu, Fe, K, Zn).

## Materials and methods

### Animal experiments

The animal experiments were performed in accordance with Polish and European regulations, and were approved by the Ethical Committee of the Medical College, Jagiellonian University in Krakow (Decision No. 129/2014). The rats were provided by the Animal Breeding Farm of the Faculty of Pharmacy, the Medical College, Jagiellonian University in Krakow. Male Wistar rats (240–260 g) were housed under standard laboratory conditions with a natural day-night cycle, a temperature of 22 °C, the humidity at 55 ± 5 %, and with free access to food and water.

The animals were randomly divided into 4 groups. For acute studies a single dose (equivalent to 6 mg Ti/kg body weight) of a solution of titanium citrate (prepared by the procedure described by Deng et al. (2004) was administered intravenously (*i.v.*) to 6 rats, and they were then euthanized 30 min (n = 3) and 180 min (n = 3) after the injection. In the multiple dose studies a group of 3 rats was given a daily dose of 6 mg Ti/kg body weight by gavage (*p.o.*) for 30 days. The control rats (n = 3) received water without titanium citrate.

At the indicated time points (acute studies) or 24 h after the last oral dose (chronic studies), the rats were deeply anesthetized with thiopental (75 mg/kg b.w., intraperitoneal injection) and euthanized. Samples of liver, kidneys and spleen were collected and immediately frozen.

### Samples preparation

For µ-SRXRF measurements the samples were mounted on the cryotome head with Shandon Cryomatrix (Thermo Electron Corporation, Pittsburgh, USA), frozen to −20 °C, cut using a Cryotome FSE cryostat (Thermo Scientific, UK) into 25  µm thick sections and mounted immediately onto a 4 µm thick Ultralene foil (SPEX, CertiPrep, USA) stretched on a PMMA ring. Afterwards, the slices were freeze-dried at −80 °C (3 days), −30 °C (1 day) and 4 °C (1 day). Then they were stored in desiccators at room temperature.

### µ-SRXRF analysis

The imaging of the elemental distribution of elements in thin freeze-dried slices of organs was done with the use of a micro synchrotron radiation-induced X-ray fluorescence (µ-SRXRF) technique. The experiment was conducted at the FLUO bending-magnet beamline of the ANKA synchrotron laboratory at Karlsruhe Institute of Technology in Karlsruhe (Germany). All measurements were carried out at a standard 45°/45° geometry. The primary beam of 10 keV photons was shaped with the use of slits—the output beam size was 250 µm (V) × 177 µm (H). Secondary radiation from the sample was collected with a KETEK silicon drift detector with a 50 mm^2^ active area. The flux of the primary beam was monitored with the use of an ionisation chamber and then used for normalisation of the acquired data. Analysed samples were mounted on a motorised X–Y–Z stage. The step size of the scan was the same in both directions and was equal to 250 µm. The average scanning area varied from 85 mm^2^ for spleen to 210 mm^2^ for liver. The counting time per pixel varied from 2 s for spleen and kidney to 3 s for liver.

### Data analysis

All spectra were processed in the batch mode of AXIL software from the QXAS package (Bernasconi et al. 1996).

The calibration of sensitivity of the spectrometer was done with the use of thin-film standards with certified mass deposit per unit area: multielemental (NIST SRM 1831) and single compound standards of KI, Cu, Fe, Ti, Zn (Micromatter, Canada). The calibration was done in order to recalculate the measured X-ray fluorescence intensity into mass deposits per unit area with the use of the external standard method. All tissue samples were treated as thin samples, which is justified for elements above chlorine for employed thickness of sections (Szczerbowska-Boruchowska et al. 2012).

The mass deposits per unit area were calculated with following formula:

$$M_{d,x,y,i} = \frac{{I_{x,y,i} }}{{I_{std,i} }}M_{d,std,i}$$

where $M_{d,x,y,i}$ is mass deposit per unit area of $i$-th element in a pixel with $x,y$ coordinates, $\,I_{x,y,i}$ is normalized X-ray fluorescence intensity if $i$-th element in a pixel with $x,y$ coordinates, $I_{std,i}$ is a X-ray fluorescence intensity if $i$-th element measured for standard sample and $M_{d,std,i}$ is certified mass deposit per unit area of standard sample.

The inter-element correlations were calculated on the basis of mass deposits per unit area, and prior to this operation all pixels outside the tissue were removed. A Pearson’s correlation analysis was applied to this dataset. Because tissue samples were treated as thin samples, correlations with Cl and elements below it (P, S) were not taken into account.

## Results and discussion

A total of 38 samples from twelve rats were analyzed. The organs of the control animals contained no titanium, which suggested that the rats were not exposed to metal from other sources.

**Fig. 1:**
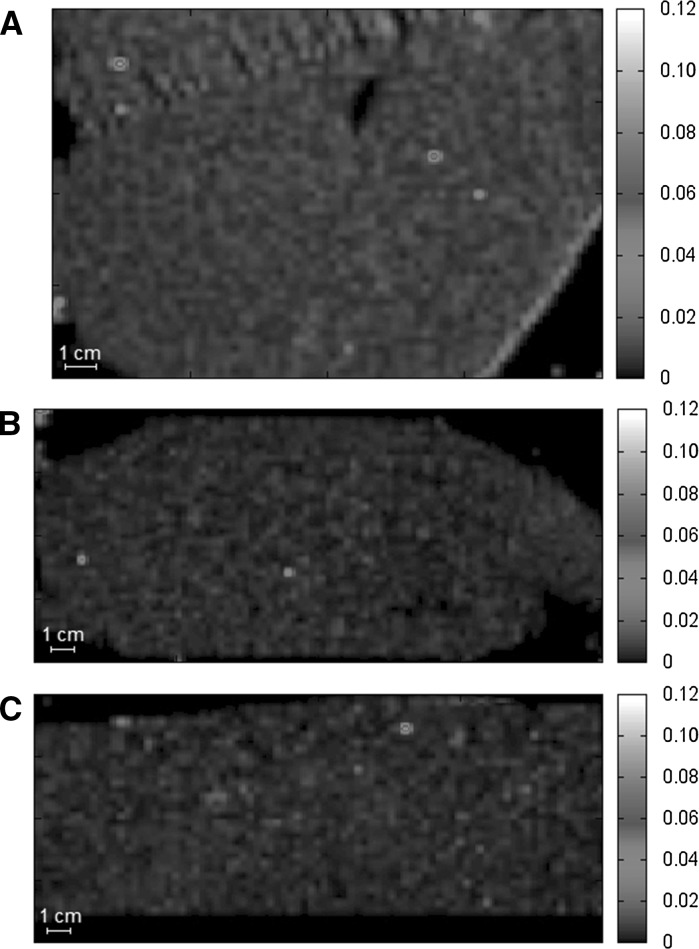
Distribution of titanium in thin sections of median (**a**), *left* lateral (**b**) and *right* lateral (**c**) lobes of rat liver at 180 min after injection of single dose of titanium citrate. Data are presented in µg cm^−2^

To begin with, three lobes of liver (median, left and right lateral) from a rat from the *i.v.* group (at 180 min) were scanned to check if the titanium distribution was different in these parts of the organ. The obtained maps of metal, presented in Fig. 1, were similar and, as such, the median lobe was chosen for further analysis.

**Fig. 2:**
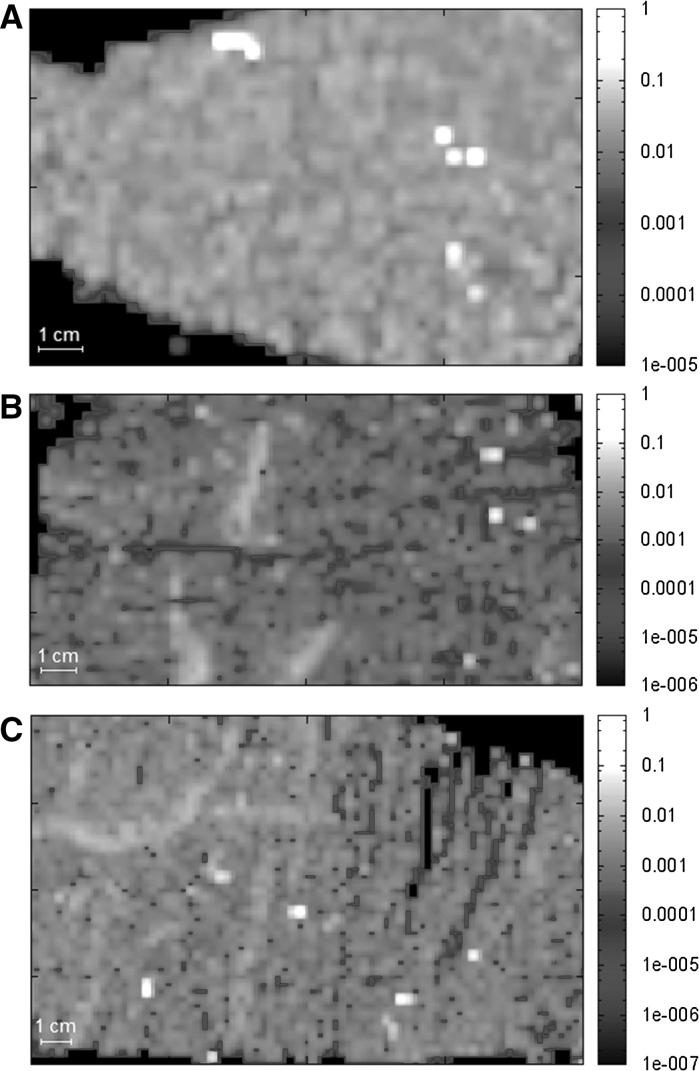
Distribution of titanium in thin sections of liver collected from animals after 30-days oral exposure to Ti (**a**), at 30 min (**b**) and 180 min (**c**) after injection of single dose of titanium citrate. Data are presented in µg cm^−2^ (log scale)

Figure 2 shows the µ-SRXRF maps of thin sections prepared from the liver of rats receiving either repeated oral (Fig. 2a) or single intravenous (Fig. 2b, c) doses of titanium citrate. The groups consisted of three animals, but only one representative map from each one was chosen. The distribution of titanium in all liver samples is not even. The single points of Ti deposition are present, especially in the organ collected from animals following long-term exposure. This group was also characterized by the highest surface mass of Ti, which indicates that metal probably accumulates gradually in the liver.

Pearson correlation study revealed a clear co-localization of Ti and Ca in livers of groups exposed to titanium. It was indicated by a statistically significant linear correlation (correlation coefficient between 0.48 and 0.80). Exemplary correlation matrix between elemental mass deposits per unit area in liver for the *i.v.* group (at 30 min) is presented in Table 1. Such a trend was not observed in the organs of the control animals (mean correlation coefficient—0.25). Some areas of liver with the accumulation of titanium were also characterized by a high amount of calcium. We assume that an unknown titanium compound may have participated in the organ (especially after long-term exposure).

**Table 1:** Pearson’s correlation matrix for mass deposits per unit area in the livers of rats at 30 min after intravenous administration of soluble Ti (n = 3)

	Ca	Cu	Fe	K	Ti	Zn
Ca	1.00					
Cu	−0.03	1.00				
Fe	0.07	0.27	1.00			
K	0.05	0.88^a^	0.37	1.00		
Ti	0.52^a^	0.12	0.39	0.26	1.00	
Zn	0.09	0.47^a^	0.21	0.43^a^	0.41^a^	1.00

^a^Statistically significant correlation coefficient, p < 0.05

**Fig. 3:**
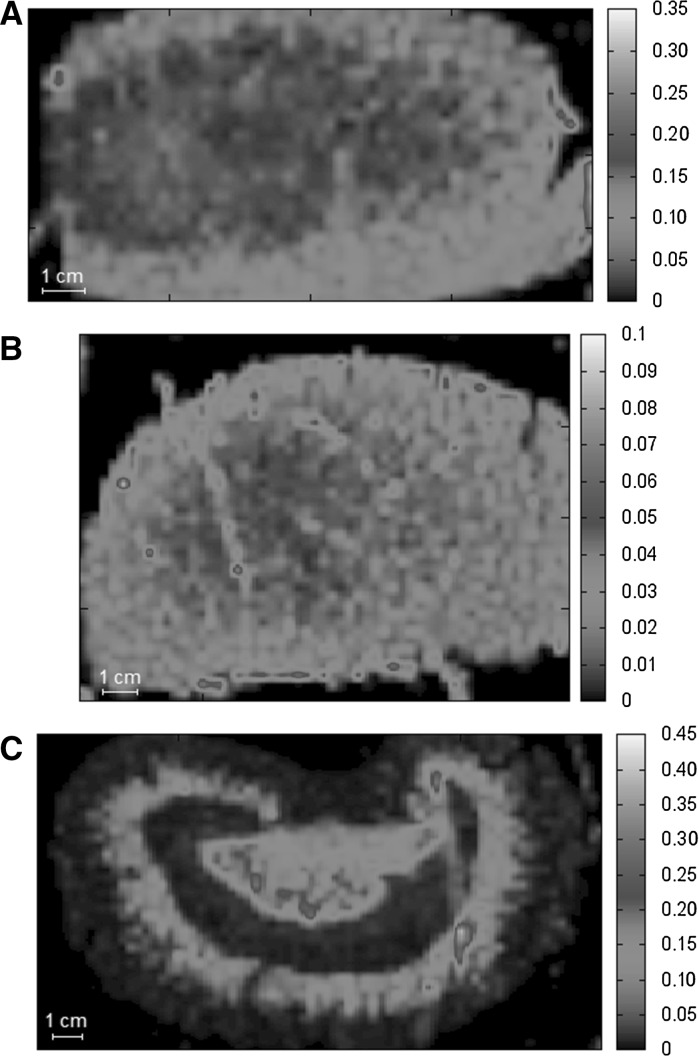
Distribution of titanium in thin sections of kidney collected from animals after 30-days oral exposure to Ti (**a**), at 30 min (**b**) and 180 min (**c**) after injection of a single dose of titanium salt. Data are presented in µg cm^−2^

The distribution of Ti in kidneys, presented on Fig. 3, is even in the organs of animals after either *p.o.* (Figure 3a) or *i.v.* (Fig. 3b, c) dosing of soluble titanium citrate. Metal accumulates in the renal cortex as a result of repeated administration of Ti, although maps obtained for both *i.v.* groups indicate that metal is quickly eliminated via the kidneys. After intravenous administration titanium is distinctly localized in the renal cortex (at 30 min) and renal pelvis (at 180 min).

The correlation analysis did not indicate the presence of a similar significant relationship between titanium and another element as in the case of liver. No linear correlation was observed between elements in any of groups.

**Fig. 4:**
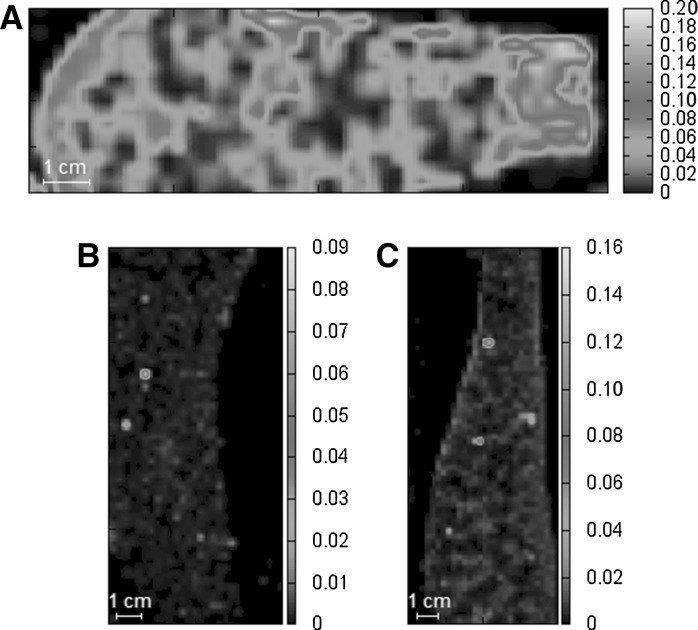
Distribution of titanium in thin sections of spleen collected from animals after 30-days oral exposure to Ti (**a**), at 30 min (**b**) and 180 min (**c**) after injection of single dose of titanium salt. Data are presented in µg cm^−2^

Maps visualizing the biodistribution of titanium in the spleen (Fig. 4) show that titanium did not accumulate in large amounts in this organ after intravenous administration. On the other hand, the long-term oral exposure to titanium salt resulted in the deposition of metal in large areas of spleen (Fig. 4a).

The numerous strong correlations between titanium and other elements were observed in spleen samples from *i.v.* group at 180 min after Ti administration (Table 2). In another *i.v.* group titanium was positively correlated with Ca (r = 0.36). In the case of the spleens of animals exposed to long-term oral administration of metal salt there was only one correlation between Ti and Fe (r = 0.36).

**Table 2:** Pearson’s correlation matrix for mass deposits per unit area in the spleens of rats at 180 min after intravenous administration of soluble Ti (n = 3)

	Ca	Cu	Fe	K	Ti	Zn
Ca	1.00					
Cu	−0.04	1.00				
Fe	0.03	0.64^a^	1.00			
K	0.04	0.54^a^	0.20	1.00		
Ti	0.43^a^	0.49^a^	0.50^a^	0.41^a^	1.00	
Zn	0.08	0.51^a^	0.11	0.92^a^	0.40	1.00

^a^Statistically significant correlation coefficient, p < 0.05

On the basis of our results it can be concluded that Ti ions accumulate mainly in kidneys, and to some extent in the liver and spleen after intravenous administration. As was mentioned before, this is the first study presenting the bioimaging of ionic titanium at the organ level, and similar experiments were conducted only with titanium dioxide nanoparticles. For example, SEM/EDS technique was used to observe the agglomerates of nanoparticles in sections of liver, kidney, lung, heart, and spleen, collected from mice after subcutaneous or intravenous injections of nano-TiO_2_ (Patri et al. 2009). However, only parts of organs were presented on elemental maps. Zhang et al. (2015) obtained quantitative maps of the distribution of TiO_2_ in lung collected from rats after intratracheal administration of nanoparticles, and observed the distinct differences in the pulmonary distribution of nano-TiO_2_ among the lobes of the lung.

Imagining techniques can also be applied in the studies of the corrosion of titanium implants. SRXRF technique was used to examine the migration of Ti ions to oral mucosa tissues close to the metal restoration (Sugiyama et al. 2015) and to the newly formed bone around the implant (Hoerth et al. 2014). Another studies on this topic utilized PIXE. Passi et al. (2002) characterized the deposits of Ti released from the dental implants to bone. They found that titanium mostly retained in the tissues around implant. Another study focused on the metallic elements from knee prosthesis that leaked into surrounding capsular tissue (Guibert et al. 2006). The debris of titanium implant penetrated the capsular tissue to a depth of about several thousand micrometers.

Due to the lack of information on the spatial distribution of Ti ions in organs, we compared our results with those from studies where the total metal content after digestion of the organs was examined. Umbreit et al. (2012) analysed the tissue distribution of nano-TiO_2_ in mice after the intravenous (56 or 560 mg kg^−1^ per mouse) injection. The mice were inspected at 2, 4, 12 and 26 weeks after administration. Over that time microgranulomas (clusters of macrophages and lymphocytes with agglomerated TiO_2_) were observed in the main filtering organs—liver, lungs and spleen. The liver plays a key role in accounting for the rapid accumulation of TiO_2_ particles after exposure via the intravenous route (Xie et al. 2011). Another study (Cho et al. 2013) demonstrated the low bioavailability of titanium in the form of orally administered TiO_2_ nanoparticles—its concentration in the liver, spleen, kidney, and brain of rats was not significantly different from results obtained for the control group. Our results indicated that soluble Ti is retained in organs after oral exposure. Overall, the distribution of the ionic form of metal seems to be different than those of titanium dioxide nanoparticles.

## Conclusions

This study investigates the biodistribution of titanium in ionic form after single intravenous or 30-days intragastric administration of a low dose of soluble metal salt on an animal model. The liver was characterized by single points of Ti accumulation, where in several cases calcium was also present in high concentration. It is presumed that some kinds of Ti compound may participate in these parts of the liver. These observations require further investigation. In kidneys metal had different concentrations in major regions: the renal cortex and renal pelvis. In *i.v.* groups most of the titanium was localized in the renal cortex (at 30 min) or renal pelvis (at 180 min), while in the *p.o.* group metal accumulated in the renal cortex. In the case of the spleen, almost no accumulation was observed in *i.v.* groups, but after oral administration metal aggregates in several regions of the organ. Accumulation of Ti in kidney, liver and spleen was observed after long-term exposure which can simulate this situation in patients with titanium implants. The results of correlation analysis suggested that titanium did not replace other elements in tissues (only several positive correlations were found). The strong association between Ti and Ca observed in liver supports the earlier presumption about the formation of unknown chemical species.

The obtained results indicate that µ-SRXRF can provide spatial distribution images to show regional deposition and accumulation of titanium in whole organs. One of the disadvantages of this technique is limited spatial resolution as well as limited range of detectable elements. It was found that ionic titanium displays a different distribution pattern to TiO_2_ nanoparticles. Our findings contribute to a first step toward the identification of potential target organs for titanium in ionic form, and could be useful during clinical examination of patients with titanium implants.
